# VAC-Stent in the Treatment of Post-Esophagectomy Anastomotic Leaks: A New “Kid on the Block” Who Marries the Best of Old Techniques—A Review

**DOI:** 10.3390/jcm13133805

**Published:** 2024-06-28

**Authors:** Giuseppe Dell’Anna, Lorella Fanti, Jacopo Fanizza, Rukaia Barà, Alberto Barchi, Ernesto Fasulo, Ugo Elmore, Riccardo Rosati, Vito Annese, Liboria Laterza, Lorenzo Fuccio, Francesco Azzolini, Silvio Danese, Francesco Vito Mandarino

**Affiliations:** 1 Gastroenterology and Gastrointestinal Endoscopy Division, IRCCS San Raffaele Institute, Via Olgettina 60, 20132 Milan, Italy; dellanna.giuseppe@hsr.it (G.D.); fanti.lorella@hsr.it (L.F.); fanizza.jacopo@hsr.it (J.F.); bara.rukaia@hsr.it (R.B.); barchi.alberto@hsr.it (A.B.); fasulo.ernesto@hsr.it (E.F.); azzolini.francesco@hsr.it (F.A.); danese.silvio@hsr.it (S.D.); 2 Gastroenterology and Gastrointestinal Endoscopy Division, IRCCS Policlinico San Donato, Piazza Edmondo Malan 2, 20097 San Donato Milanese, Italy; vito.annese@grupposandonato.it; 3 Faculty of Medicine and Surgery, Vita-Salute San Raffaele University, Via Olgettina 56, 20132 Milan, Italy; rosati.riccardo@hsr.it,; 4 Gastrointestinal Surgery Unit, IRCCS San Raffaele Institute, Via Olgettina 60, 20132 Milan, Italy; elmore.ugo@hsr.it; 5 Unit of Gastroenterology, Department of Medical and Surgical Sciences, S. Orsola-Malpighi University Hospital, University of Bologna, Via Massarenti 9, 40138 Bologna, Italy; liboria.laterza@gmail.com (L.L.); lorenzofuccio@gmail.com (L.F.)

**Keywords:** anastomotic leak, esophagectomy, endoscopic vacuum therapy, self-expandable metal stent, VAC-Stent

## Abstract

Esophagectomy, while a pivotal treatment for esophageal cancer, is not without adverse events. Among these, anastomotic leak (AL) is the most feared complication, threatening patient lives and incurring significant healthcare costs. The management of AL is complex and lacks standardization. Given the high morbidity and mortality rates associated with redo-surgery, which poses risks for already fragile patients, various endoscopic treatments have been developed over time. Self-expandable metallic stents (SEMSs) were the most widely used treatment until the early 2000s. The mechanism of action of SEMSs includes covering the wall defect, protecting it from secretions, and promoting healing. In 2010, endoscopic vacuum therapy (EVT) emerged as a viable alternative for treating ALs, quickly gaining acceptance in clinical practice. EVT involves placing a dedicated sponge under negative pressure inside or adjacent to the wall defect, aiming to clear the leak and promote granulation tissue formation. More recently, the VAC-Stent entered the scenario of endoscopic treatment of post-esophagectomy ALs. This device combines a fully covered SEMS with an integrated EVT sponge, blending the ability of SEMSs to exclude defects and maintain the patency of the esophageal lumen with the capacity of EVT to aspirate secretions and promote the formation of granulation tissue. Although the literature on this new device is not extensive, early results from the application of VAC-Stent have shown promising outcomes. This review aims to synthesize the preliminary efficacy and safety data on the device, thoroughly analyze its advantages over traditional techniques and disadvantages, explore areas for improvement, and propose future directions.

## 1. Introduction

Esophageal cancer remains a leading cause of cancer-related deaths worldwide. Recent advancements have led to increasingly multidisciplinary treatment approaches, with a significant focus on neoadjuvant therapies. However, surgery remains the primary treatment option for curative intent [1,2]. Despite its potential, esophageal surgery is associated with significant morbidity and substantial postoperative complications [3]. Among these, anastomotic leak (AL) is particularly concerning due to its high morbidity and mortality risk, resulting in prolonged hospital stays and increased healthcare costs [3,4].

In 2015, the Esophagectomy Consensus Group (ECCG) defined AL as “a full-thickness wall defect involving the esophagus, anastomosis, suture line, or conduit”, further categorizing it into three severity levels [5,6]. Despite progress in surgical techniques, large cohort studies have shown that the incidence of post-esophagectomy leaks remains high, exceeding 10% [7,8,9,10,11]. This persistent challenge underscores the critical need for effective management and treatment strategies to mitigate the risks associated with AL.

The treatment strategy for AL is not standardized. The choice of management approach depends on several factors, including the timing of its diagnosis, the patient’s overall clinical condition, and the presence of necrosis or ischemia in the conduit [3,12]. Surgical reintervention is generally reserved for emergent cases or those involving severe sepsis and/or conduit necrosis. However, re-surgery carries high morbidity and mortality due to the invasiveness of the procedure and the often-critical conditions of the patient. In non-septic and non-emergency scenarios, endoscopic treatment is the preferred approach. Over the years, numerous endoscopic techniques have been developed and refined, driven by both growing scientific interest and technological advancements in the field.

Self-expanding metal stents (SEMSs) and endoscopic vacuum therapy (EVT) stand out as the primary endoscopic treatments for post-esophagectomy ALs. Other endoscopic options include metallic clips (through-the-scope-clips (TTSCs) and over-the-scope clips (OTSCs)); sealants, like cyanoacrylates; and suturing systems, such as the overstitch system by Apollo Endosurgery, Inc (Boston Scientific, Malborough, USA). [13,14,15].

SEMSs have been a mainstay in treatment since the early 2000s. The primary mechanism of action of SEMSs is to provide a physical barrier that blocks the exposure of internal tissues to saliva and other potentially harmful secretions. This promotes the healing of the dehiscence. However, a significant issue related to SEMSs is migration, which can lead to reduced efficacy of the treatment, potential re-exposure of the leak, and may require additional interventions to reposition or replace the stent.

EVT, developed in 2008, was inspired by vacuum therapy techniques used in plastic surgery to treat skin wounds. In recent years, growing evidence has established EVT as the gold standard for the treatment of post-esophagectomy ALs. EVT involves placing sponges that generate negative pressure within the lumen or associated cavities to aspirate secretions, thereby promoting the formation of granulation tissue and facilitating the healing of leaks. However, a significant limitation of EVT is the necessity for closely timed procedures to replace the sponges, which can increase the overall treatment burden for patients, potentially leading to greater discomfort and higher healthcare costs [16,17,18,19].

The VAC-Stent, developed by Microtech Endoscopy (Dusseldorf, Germany), represents the latest innovation in the field of endoscopic treatment for ALs. This device is a fully covered nitinol SEMS that incorporates a polyurethane sponge on its body, thereby combining the benefits of SEMSs and EVT. Although the data on its application are still preliminary, there is burgeoning interest in the VAC-Stent, as it promises a more integrated approach to sealing leaks [20,21].

This narrative review aims to synthesize the emerging evidence on the VAC-Stent, discuss its indications, and consider its potential role in the management of ALs following esophagectomy. We also intend to highlight the potential advantages and disadvantages of the VAC-Stent in comparison with established treatments, such as SEMSs and EVT

## 2. Main Endoscopic Treatments of Anastomotic Leaks

### 2.1. Self-Expandable Metal Stents (SEMSs)

Over the past two decades, SEMSs have been the primary treatment for upper gastrointestinal (GI) ALs and wall defects [16]. According to guidelines from the European Society of Gastrointestinal Endoscopy (ESGE), SEMSs are recommended for the treatment of GI leaks, fistulas, and defects larger than 20 mm, although no specific stent design is mandated [22,23,24].

Currently, the two most commonly used SEMS models for treating post-esophagectomy AL are fully-covered SEMSs (FC-SEMSs) and partially-covered SEMSs (PC-SEMSs). Previously, self-expandable plastic stents (SEPS) were also employed, but their use has declined due to SEMSs demonstrating superior efficacy [16,25,26,27]. FC-SEMSs feature a plastic or silicon layer that completely covers the metal mesh, while PC-SEMSs have their proximal and distal ends uncovered. This design aims to prevent migration but may promote mucosal ingrowth. Both FC-SEMSs and PC-SEMSs have demonstrated acceptable efficacy in the treatment of esophageal ALs [17,28,29].

**Figure 1:**
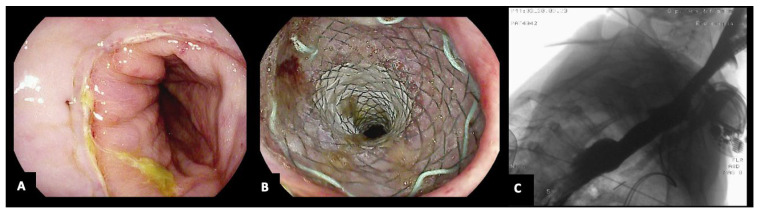
A 75-year-old woman with post-Ivor-Lewis esophagectomy anastomotic leak (**A**), treated by FC-SEMS (**B**,**C**). The copyright of the image belongs to the authors.

SEMSs are deployed under radiological guidance after a guidewire is positioned under endoscopic visualization. Within 24 to 48 h, the metal mesh of the SEMS expands radially to its full diameter, adhering to the mucosal wall. This expansion enables the SEMS to effectively protect the defect from luminal contents such as saliva, secretions, and digestive enzymes, thus promoting healing (Figure 1). Additionally, SEMSs permit the resumption of oral intake, which is crucial in the postoperative period for typically malnourished patients [16,17,29,30]. This capability can significantly enhance their nutritional status and overall recovery.

Although current guidelines do not specify the exact timing, SEMSs are typically removed after 6–8 weeks [22].

In published studies, clinical success (CS) rates for SEMSs in sealing post-esophagectomy ALs vary significantly, with data ranging from 63.5% to 100%. However, these findings must be interpreted with caution due to small sample sizes, enrollment biases, and the diversity of patient populations included [16,28,29,30,31,32,33].

In a study conducted by Anderloni et al., which enrolled 49 patients with post-esophageal surgery AL who underwent SEMS placement, the overall CS rate was 60.5%, with no differences between FC-SEMSs and PC-SEMSs (57.1% and 64.7%, respectively). The overall adverse events (AEs) rate was 38.8%. Overall stent migration rate was 8.16%: 7.14% in the FC-SEMSs cohort and 8.6% in the PC-SEMS cohort [34].

Similarly, another study by Plum et al., involving 70 patients with post-Ivor Lewis esophagectomy ALs treated with FC-SEMSs, reported a CS rate of 70% and a median treatment duration of 28 days (range 7–87). Twenty patients (28.6%) experienced stent-related AEs, with stent dislocation being the most frequent (18.6%). All were resolved endoscopically by replacing the stent with a new one [35].

Recently, customized FC-SEMSs with an outer double layer (anti-migration device) have been introduced to reduce the risk of migration. An example of this is the Niti-S™ Beta™ Esophageal Stent developed by Taewoong Medical (Seoul, Republic of Korea). An additional advantage of this stent is its large diameter, which reduces stent leakage—defined as liquid infiltration at the stent edges, often a cause of failure, as it impairs the healing process.

A study by Segura PS et al. included 23 patients with post-esophageal surgery ALs and esophageal perforations treated with Niti-S™ Beta™ Esophageal Stents. The authors reported a CS rate of 75% (17/23 patients). FC-SEMS migration was observed in 21.7% of cases [36].

The largest series evaluating double-layer FC-SEMSs comes from our institution. The study involved 37 patients with post-esophagectomy AL, treated with a total of 75 FC-SEMS (2.0 ± 1.3 FC-SEMSs for patients). The closure of the leak or defect was achieved in 23 patients (62.2%). Migration was observed in 17/75 cases (22.7%). Interestingly, previous neoadjuvant therapy (OR 9.3, *p* = 0.01), fistula (OR 6.5, *p* = 0.01), and stent leak (OR 17.01, *p* = 0.01) were statistically associated with the failure of FC-SEMS treatment [29].

SEMS migration remains a feared complication even with these new designs of FC-SEMS, and further research is needed to determine the best type of SEMS that can reduce the incidence of this AE while simultaneously ensuring treatment efficacy.

In recent years, fixation techniques have been tested to prevent SEMS migration. These include TTSC [37], OTSC [38], and endosuturing devices.

Papaefthymiou A et al. recently conducted a comprehensive meta-analysis of 10 studies involving 1014 patients to assess the success rate of fixation systems for esophageal SEMSs, including endoscopic suturing, OTSC, and TTSC. The analysis revealed a significantly lower rate of migration in the fixation group (OR 0.20, *p* = 0.01). Additionally, no significant differences were found in the AE rates between the fixation and control groups (*p* = 0.06) [37].

### 2.2. Endoscopic Vacuum Therapy (EVT)

**Figure 2:**
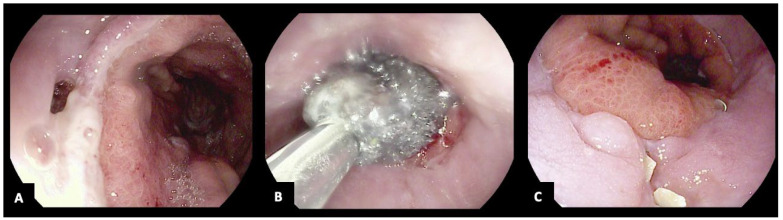
A 53-year-old woman with an esophageal anastomotic leak following Ivor Lewis esophagectomy (**A**) treated with intraluminal sponge endoscopic vacuum therapy (**B**,**C**). The copyright of the image belongs to the authors.

EVT has become a primary treatment for post-esophagectomy AL. It was initially employed 15 years ago in the colon for treating ALs with associated collections, later expanding its application to the esophagus and upper GI tract [18,39] (Figure 2).

EVT involves the placement of a polyurethane sponge connected via a tube to a vacuum device. This device applies constant negative pressure, which can be adjusted on the basis of individual cases [40]. The recommended pressure is cited as 125 mmHg, although it lacks definitive consensus or established guidelines. The vacuum aids in healing by removing inflammatory and infected secretions and enhancing tissue perfusion of the AL, which facilitates granulation tissue formation.

The sponge typically needs to be changed every 3 to 5 days. This frequency ensures that the sponge continues to effectively manage secretions and support granulation tissue formation without overstaying, which could lead to potential complications, such as infection or adherence to the tissue. Regular replacement is crucial for maintaining the efficacy of the vacuum therapy and for adjusting the treatment as the patient’s condition progresses.

EVT can be performed using either an intracavitary or intraluminal placement of the sponge, depending on the specifics of the defect. The intracavitary method involves placing the sponge inside the cavity associated with the leak for direct aspiration and is typically preferred for larger defects with fluid collections. Conversely, the intraluminal method positions the sponge within the organ’s lumen, straddling the wall defect, often at the anastomotic site, and is suitable for smaller defects that lack associated cavities [18,30,39,41]. However, there are currently no data in the literature that directly compares the efficacy of these two techniques.

The commercial model of EVT is Esosponge (Braun, Aesculap AG, Tuttlingen, Germany). However, EVT can also be manually set up, with a sponge attached with sutures to the distal end of a suction catheter connected to a vacuum pump.

In recent years, extensive research has solidified the role of EVT as a game-changer approach for managing post-esophagectomy AL. Recent retrospective series have reported CS rates ranging from 90% to 100% [42,43,44,45]. However, it is important to note that these studies often involve small cohorts, frequently fewer than 20 patients, which may limit the generalizability of the results.

In a multicenter retrospective series by Jung et al., which included 119 patients predominantly affected by post-esophagectomy AL (110/119 cases, 92.4%), EVT demonstrated clinical efficacy in 84 patients (70.6%). The total mean number of EVT procedures was 3.93 (1–19) and 4.20 (1–23) for the CS group and clinical failure group, respectively. Neoadjuvant treatments and intraluminal methods were identified as independent predictors of clinical failure. AEs occurred in 10.9% of cases, with sponge dislocation being the most frequent [46].

In a recent study by 19 Spanish hospitals involving 102 patients (89 with AL), EVT led to defect closure in 84 cases (82%). The time from diagnosis of the defect to the initiation of EVT was the only independent predictor of treatment failure (OR 1.03, *p* = 0.005) [47].

In another study of 38 patients with post-esophagectomy ALs (81% after Ivor Lewis esophagectomy), EVT showed efficacy in 74% of cases. The median number of EVT-related endoscopies was 4 (range 3–8). Two severe AEs EVT-related were documented: a tracheoesophageal fistula and a defect expansion caused by the over-tube during sponge replacement, both requiring surgical intervention [48].

In a recent multicentric study by Luttikhold et al. enrolling 27 patients, EVT was used for the treatment of iatrogenic perforations. The authors reported a CS rate of 89% and an AE rate of 7%. These included one case of defect enlargement due to scope passage during sponge removal and one case of moderate bleeding at the sponge site requiring blood transfusion [49].

EVT has proven to be an effective rescue therapy for post-esophagectomy ALs. In a recent series by the San Raffaele group, which included 12 patients with post-esophagectomy AL previously treated with ineffective redo-surgery (25%) or other endoscopic treatments (75%), EVT led to the closure of the defect in 9 patients (75%). Only one case of migration of the sponge (1.7%) was reported as an AE. During follow-up, one long-term stenosis (1.7%) was observed and successfully treated endoscopically [19].

A new frontier in EVT is its preemptive application during esophagectomy to prevent AL.

In a preliminary study conducted by Muller et al., 67 patients underwent pre-emptive EVT sponge placement. Of these, 49 patients (73%) experienced regular healing of the anastomosis. In the remaining 18 patients (27%), EVT was extended after the removal of the prophylactic sponge due to worrisome endoscopic signs of anastomosis. Among them, 13 patients (72.2%) showed regular healing of the anastomosis, 4 patients (22.3%) developed Als—which were treated with additional cycles of EVT—and one patient required surgery due to signs of conduit necrosis. The overall AL rate after pre-emptive therapy was 7.5% [50].

From these initial pieces of evidence, prophylactic EVT appears to be a safe procedure with the potential to reduce the rate of post-esophagectomy ALs. It can promote the healing of minimal defects in the anastomosis immediately after surgery and treat postoperative anastomotic ischemia, thus favoring the formation of granulation tissue. Furthermore, some authors have suggested that the presence of the sponge in the esophageal lumen may reduce the risk of aspiration pneumonia during the immediate postoperative period [50].

More robust data on preemptive EVT will come from the ongoing randomized controlled trial (the preSPONGE trial, NCT04162860) [51].

### 2.3. Comparison between SEMSs and EVT

Current studies comparing EVT and SEMSs in the treatment of upper GI defects are methodologically poor due to their retrospective nature, small cohort sizes, and heterogeneous inclusion criteria [16,17,30].

In the largest cohort study published to date by Berlth et al. enrolling 111 patients with post-oncological gastroesophageal surgery ALs (83.8% after esophagectomy)—76 in the SEMSs group and 35 in the EVT group—the AL closure rate was higher in the EVT group than in the SEMS group, although not statistically significant (85.7% versus 72.4%, *p* = 0.152, respectively). Despite the shorter treatment duration of EVT compared to SEMS (12 (3–58) days vs. 27 (1–152) days; *p* < 0.001), no significant difference was observed in the length of hospital stay (*p* = 0.812). Additionally, the AE rate was similar between the groups (26% SEMSs vs. 15% EVT; *p* = 0.614) [52].

To address the biases of previous studies, our group recently conducted a matched case–control study comparing EVT and FC-SEMSs (Niti-S™ Beta™ Esophageal Stent Taewoong Medical, Seoul, Republic of Korea) in the treatment of ALs < 30 mm following Ivor Lewis esophagectomy. Controls were matched in a 1:1 ratio according to age, body mass index (BMI), and AL size, with 22 patients per arm. The CS rate was found to not be different between the two techniques (EVT 90.9% vs. SEMSs 72.7%, *p* = 0.11, respectively). Remarkably, the SEMS group exhibited a higher rate of migrations (15.3% vs. 1.6%, *p* = 0.0001) [53].

According to this evidence, we can hypothesize that for small defects (<30 mm) not associated with cavities where EVT is positioned intraluminally, EVT and SEMS may have similar efficacy. In such scenarios, the choice between EVT and SEMS might hinge on factors such as procedural preferences, patient-specific considerations, and the experience of the medical team.

Two recent systematic reviews and meta-analyses have aimed to synthesize the results of the studies currently published on EVT vs. SEMSs in the treatment of upper GI defects.

In a meta-analysis by Scognamiglio et al. published in 2020, which included five studies and 274 patients with AL following esophageal surgery, EVT was found to be significantly associated with a higher rate of AL closure (OR 3.14, 95% CI 1.23 to 7.98), a higher number of endoscopic devices used (pooled median difference of 3.09; 95% CI 1.54 to 4.64), a shorter treatment duration (pooled median difference −11.90 days; 95% CI −18.59 to −5.21 days), and a lower mortality rate (OR 0.39, 95% CI 0.18 to 0.83) compared with SEMSs [54].

A more recent meta-analysis by Mandarino FV et al., including 8 studies and a total of 357 patients with post-esophagectomy or gastrectomy ALs treated with EVT or SEMS, broadly confirmed the results of the previous one [12]. EVT showed a higher CS rate (OR 2.58, 95% CI 1.43–4.66), a higher number of devices used (pooled mean difference 4.90, 95% CI 3.08–6.71), a shorter treatment duration (pooled mean difference −9.18, 95% CI −17.05–−1.32), lower short-term AEs rates (OR 0.35, 95% CI 0.18–0.71), and a lower mortality rate (OR 0.47, 95% CI 0.24–0.92) compared with SEMSs. However, in a subgroup analysis that included only studies enrolling patients with AL after oncologic surgery, no significant difference in CS rate was observed between the two techniques (OR 1.59, 95% CI 0.74–3.40, I2 = 0%) [12].

Currently, a phase II randomized controlled trial (ESOLEAK trial; NCT03962244) comparing EVT and SEMSs in the treatment of AL following Ivor Lewis esophagectomy is ongoing, which is expected to provide more robust evidence on the subject [55].

## 3. VAC-Stent

### 3.1. Design of the VAC-Stent

**Figure 3:**
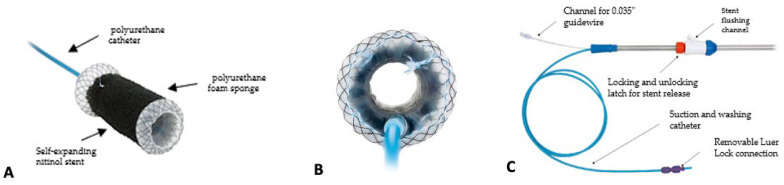
VAC-Stent. (**A**) Composition of VAC-Stent; (**B**) view into the stent; (**C**) the complete VAC-Stent GI system. The copyright of the image belongs to the authors.

The VAC-Stent combines elements of both SEMSs and EVT into a single device. It includes a nitinol FC-SEMS with a silicone membrane and an expanded polyurethane sponge wrapped around it. A polyurethane catheter, 2.5 m long and 10 French in diameter, equipped with two connectors (a Luer lock and a Y-connector), connects the stent to the vacuum therapy pump (Figure 3).

The FC-SEMS has a body 50 mm long with a diameter of 14 mm and two dumbbell-shaped flanges, each 10 mm in length and 30 mm in diameter, giving a total stent length of 70 mm. The stent body incorporates six radiopaque titanium markers to assist in positioning during deployment. The sponge is attached to the FC-SEMS body by a suture thread.

The VAC-Stent comes pre-mounted on a 1-m-long over-the-wire insertion system. This system features a soft silicone distal end with a diameter of 14 mm and includes two radiopaque markers.

Stent deployment follows the steps of a standard esophageal SEMS. After the endoscopic assessment and distal placement of a 0.035-inch guidewire in the GI lumen beyond the leak, the endoscope is withdrawn. The stent release system is then advanced over the guidewire, guided by both fluoroscopy and endoscopy, using a slim endoscope (8 mm outer diameter) positioned parallel to the stent catheter [21,56,57,58,59]. After deployment, irrigation through the Y-catheter with approximately 20 cc of saline solution (0.9% NaCl) is proposed to aid in expanding the open-cell polyurethane sponge [56,57].

Currently, there are no strict guidelines regarding suction negative pressure; however, the manufacturer recommends initially setting the pump at −125 mmHg for the first 12–24 h to facilitate tissue adaptation, followed by an adjustment to between −85 and −100 mmHg. Available studies have applied continuous suction with a negative pressure ranging from −65 to −125 mmHg [20,21,56,58,60].

For stent removal, it is advised to switch off the pump 4 to 6 h beforehand. During the removal process, irrigating the sponge with saline solution through the Y-shaped connector can facilitate detachment from the esophageal wall. Rat-tooth forceps may be used to grasp the stent while simultaneously pulling the suction catheter [56,61]. In a case report, Pattynama LMD et al. reported the use of a tapered hood distal attachment cap for gentle stent removal [59].

The recommended replacement interval for each single device is 3 to 7 days.

### 3.2. Indications

**Figure 4:**
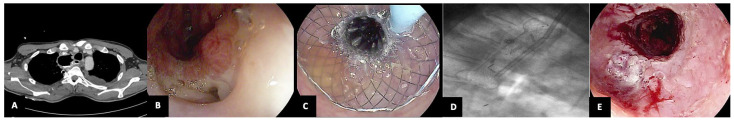
A 68-year-old patient with esophageal anastomotic leak after Ivor Lewis esophagectomy (**A**,**B**) was treated successfully with VAC-Stent placement (**C**–**E**). The copyright of the image belongs to the authors.

Post-esophagectomy ALs are the primary indication for VAC-Stent (Figure 4) [15,20].

However, due to its recent introduction and limited published evidence, the VAC-Stent has yet to establish a defined role in the treatment algorithm [23,24,62]. The mechanism of action and the device’s design suggest that the VAC-Stent should be considered, particularly in cases not associated with large cavities [56,60]. Additionally, the VAC-Stent has been investigated for prophylactic use during esophagectomy in high-risk patients to prevent AL [58].

Esophageal perforations, including iatrogenic and spontaneous cases (Boerhaave’s syndrome), are another reported indication [20]. Successful treatment of leaks following bariatric surgery, such as sleeve gastrectomy or Roux-en-Y gastric bypass, has also been documented with the VAC-Stent [63]. The treatment of leaks in the lower GI tract has only been described in a case report [64].

### 3.3. Outcomes

Since their introduction, published studies on VAC-Stent have involved nearly 100 patients. In this section, we summarize the efficacy and safety data from the major series (Table 1).

#### 3.3.1. Efficacy

The first reported use of the VAC-Stent dates to 2020 and involved a 61-year-old male patient with an esophago-jejunal anastomosis AL following a total gastrectomy. Initially, the team attempted to manage the AL with an OTSC, which proved ineffective. After the patient’s clinical conditions deteriorated and he developed sepsis, the OTSC was removed, and the first VAC-Stent was placed. Two days later, an improvement in the AL was observed endoscopically, leading to the placement of a new VAC-Stent. Four days afterward, the VAC-Stent was removed, showing complete healing of the dehiscence. The patient was discharged after a 23-day hospital stay [65].

In 2021, Lange J et al. published a case series involving three patients successfully treated with VAC-Stent. The first patient, affected by post-esophagectomy AL, underwent treatment with two sequential VAC-Stents for 12 days. The second patient, who had an esophageal perforation, had been initially unsuccessfully treated with an FC-SEMS and EVT before starting treatment with the VAC-Stent, which led to clinical success after just one session. The third patient, affected by an iatrogenic esophageal perforation caused by the removal of the LINX Reflux Management System, was successfully treated with one VAC-Stent [57].

A larger initial dataset was provided by a study at the University Hospital Cologne in 2021, which enrolled 10 patients treated with VAC-Stent for upper GI leaks, including ALs and esophageal perforations. Half of these patients had previously undergone endoscopic therapy. CS was achieved in 70% of patients, without the need for further interventions. When VAC-Stent was used as a first-line treatment, the CS rate was 80%, while as a second-line treatment, the success rate dropped to 60%. VAC-Stent was ineffective in three cases. In two cases, the rescue strategy involved EVT. Another patient diagnosed with Boerhaave’s syndrome, deteriorating clinically with septic shock, necessitated an urgent total esophagectomy [61].

In 2022, the same group published the results of a prospective trial that included 20 patients (24 VAC-Stents) who received VAC-Stent treatment. CS was achieved in 12 patients (60%). Specifically, when VAC-Stent was used as a first-line treatment, the CS rate was 71% (12/17). In cases in which VAC-Stent was not effective (8/20 patients, 40%), a second-line treatment was attempted; seven patients were successfully treated with EVT, and the other patient underwent surgical repair [60].

Another prospective series from a tertiary referral center in the Netherlands involved 10 patients with esophageal leaks, predominantly ALs, treated with VAC-Stents. In five cases, VAC-Stents were used as rescue therapy following initial EVT, and in the remaining five, they were used as a first-line therapy. CS was achieved in all cases (10/10, 100%) [21].

In 2023, Lange J et al. published the first prospective multicenter open-label study involving 15 patients with esophageal leaks, totaling 41 VAC-Stent placements. CS was observed in 12 patients (80%) after an average of 2.7 VAC-Stents per patient. Among those experiencing clinical failure, leaks underwent re-surgery in two cases, while in another case healed spontaneously after VAC-Stent removal [56].

A single-center pilot study assessed the feasibility and efficacy of preventive VAC-Stent placement in patients who underwent Ivor Lewis esophagectomy with high-risk anastomoses after neoadjuvant therapy. The primary endpoint of the study was the intraoperative technical feasibility and successful coverage of the anastomosis by the VAC-Stent. Secondary endpoints included AL rate, postoperative morbidity, and mortality. A total of 11 VAC-Stents were used for 9 patients. One patient developed an AL (1/9, 11.1%), which was successfully treated by placing two additional VAC-Stents over a total of 14 days. Complete endoscopic healing of all the anastomoses was observed (100%). The effective coverage of the esophageal anastomosis was achieved in all cases (9/9, 100%) [58].

Recently, Lange J et al. assessed the efficacy of VAC-Stent in a population of 50 patients, mostly affected by esophageal ALs (40/50, 80%), while the remaining 10 (20%) had VAC-Stents placed prophylactically. The study included three cohorts: two from previously published studies and one from an open-label multicenter registry study. A total of 92 VAC-Stents were placed, with an average of two stents per patient and an average dwell time of 5.2 days. The VAC-Stent was effective in 38 patients (76%). For patients with esophageal leak, CS was observed in 28 patients (70%), with an average of two stents (1–9) used [20].

In a case report by Shah et al., the VAC-Stent was effective in managing an esopleural fistula associated with empyema following a Roux-en-Y gastric bypass. Previous attempts to cover the defect using PC-SEMS and internal endoscopic drainage with double pigtail plastic stents were ineffective. A VAC-Stent was then positioned to cover the esopleural fistula while simultaneously clamping the pleural drainage to promote negative pressure. The VAC-Stent was removed after one week, showing evidence of complete fistula closure [63].

Pattynama et al. described a case of Boerhaave’s syndrome with a purulent cavity extending into the mediastinum treated with a VAC-Stent. After surgical revision and 60 days of EVT proved ineffective, the decision was made to initiate VAC-Stent treatment. After 14 days of therapy with two consecutive VAC-Stents, the defect was completely closed [59].

VAC-Stent also proved effective in treating an anastomotic leak in the lower GI tract of a patient who had undergone colostomy reversal following an emergency sigmoidectomy for acute diverticulitis [64].

#### 3.3.2. Safety

VAC-Stent placement is considered a safe procedure with infrequent major complications. In their initial study, Chon SH et al. reported three instances (30%) of incomplete stent removal on the first attempt due either to esophageal tissue ingrowth into the sponge or a lack of a fixation system between the sponge and the stent body, which left the sponge attached to the esophageal lumen and required separate mobilization for removal. They also documented that the internal diameter of all VAC-Stents placed never reached the maximum expansion of 14 mm, necessitating pneumatic dilation in all cases [62]. A similar issue was observed in a subsequent prospective study by the same group [60].

In a study by Lange J et al., a 7% incidence of VAC-Stent migration or dislocation was reported [56]. In this study, the authors also reported nine cases (22%) of erosion/ulceration at the stent site after removal and five cases (12%) of local bleeding, but none of these cases required additional treatment. Thirty days after discharge, a patient with dysphagia was diagnosed with an anastomotic stricture [56].

When used preventively in patients undergoing Ivor Lewis esophagectomy, Lange et al. did not observe any AEs [58].

Pattynama LMD et al. described a single case of anastomotic stricture resolved by endoscopic pneumatic dilation in a patient treated with EsoSponge and VAC-Stent following an AL at the site of the cervical anastomosis [21].

In the largest series by Lange J. et al., only three cases of minor mucosal bleeding from moderate erosions near the stent beads were observed. These issues did not lead to significant bleeding or require intervention. No severe complications were associated with the VAC-Stent in any of the 92 applications [20].

### 3.4. VAC-Stent versus Current Techniques: Advantages and Disadvantages

Given its recent development, comparative studies between the VAC-Stent, SEMSs, and EVT do not yet exist. This section aims to explore the advantages and disadvantages of the VAC-Stent compared with SEMSs and EVT individually (Table 2).

Understanding these differences is essential to determine where the VAC-Stent stands regarding clinical efficacy and identify potential areas for improvement in future designs.

#### 3.4.1. VAC-Stent vs. SEMS

##### Advantages

The main advantage of the VAC-Stent over traditional SEMS lies in its integration of vacuum therapy, which enhances the drainage and aspiration of fluid collections associated with ALs. This is particularly valuable for managing infected fluids, as it allows microbiological analysis and the administration of targeted antibiotic therapy—a process that would typically require a percutaneous drain with SEMS. Additionally, the VAC-Stent’s negative pressure is effective in healing defects by reducing tissue edema and improving blood flow and oxygenation, thus promoting granulation tissue formation [18,66].

Another significant benefit of the VAC-Stent is its innovative design. Unlike conventional SEMS, the VAC-Stent’s cylindrical body has a more flexible attachment between the inner silicone membrane and the nitinol mesh, allowing it to better conform to the anatomical site’s contours and movements. This adaptability can lead to the formation of longitudinal folds in the silicone membrane, which protrude into the lumen without impairing the stent’s functionality [56]. Without this mechanism, traditional SEMS are less adaptable to the esophagus. Due to this and the larger size, their use has been linked to developing mucosal erosions and ulcers, which are more frequent in patients undergoing neoadjuvant chemoradiotherapy or when SEMS treatment is prolonged [67,68]. Cases involving erosion into major vessels, such as the aorta or pulmonary artery, which often lead to fatal events, have also been reported [16,17]. The VAC-Stent, offering similar defect protection but with smaller dimensions and a morphology better suited to the anatomical site, is less likely to be associated with such AEs. Moreover, thanks to the negative pressure-mediated adherence of the stent body, it is less prone to stent leak.

Despite the lack of direct comparative studies, the VAC-Stent appears to be associated with a significantly lower migration rate than conventional SEMSs. This stability is attributed to three factors. The first is the complete adhesion of the stent to the esophageal walls through EVT’s negative pressure, which creates a vacuum effect that secures the stent more firmly against the tissue. The second is the anchored drainage tube acts as a stabilizing anchor; this mechanically secures the stent in place, providing additional resistance to movement caused by normal peristalsis or external physical activity. The third is the 30 mm in diameter dumbbell-shaped flare ends enhance stent anchorage [60]. For these same reasons, it is likely that the VAC-Stent is also less prone to fluid leakage between the stent and the esophageal walls.

The VAC-Stent has demonstrated efficacy as a rescue strategy in complex cases where other endoscopic treatments (SEMS, EVT) or even salvage surgery have failed [58,62]. This contrasts with the limited evidence supporting the use of SEMSs in such scenarios, which often require drainage capability in the case of resistant collections.

Furthermore, in cases of ALs associated with large anastomotic dehiscence, sequential vacuum therapy—initially placing intracavitary EVT followed by an intraluminal VAC-Stent—has the potential to ensure continuous drainage throughout the treatment. This dual vacuum therapy approach would not be feasible with traditional SEMSs.

##### Disadvantages

Despite its benefits, the VAC-Stent has some disadvantages compared with traditional SEMSs.

While difficulties in placing the VAC-Stent have not been reported in current studies, its novel release mechanism may require specialized training for both endoscopists and nurses, even those experienced in luminal stenting. Additionally, the unique design of the VAC-Stent necessitates careful coordination between medical staff to ensure proper setup and maintenance, potentially extending procedure times and increasing resource utilization.

Patients treated with SEMSs, if clinically stable, can be discharged and readmitted for endoscopic reassessment [16,17]. By contrast, the VAC-Stent requires connection to an external vacuum pump, necessitating constant monitoring by healthcare personnel. This poses challenges to patient mobility and discharge planning, and it also places additional demands on hospital resources and staffing.

The VAC-Stent requires more frequent replacement, with an average interval of 5–7 days, significantly shorter than the duration for traditional SEMS [20]. This frequent replacement increases the procedural burden and enhances the costs. Furthermore, the need for repeated interventions may lead to increased discomfort for the patient and a higher risk of complications related to the endoscopic procedure and sedation. However, this aspect should not entirely be considered a disadvantage of the VAC-Stent, as it allows for close clinical and endoscopic monitoring of AL, which could have a significant impact on subsequent management.

Another advantage of SEMSs is that patients do not experience the discomfort associated with a nasally protruding tube, a common issue with the VAC-Stent. The absence of nasal discomfort contributes to a better quality of life for patients undergoing treatment with SEMSs.

A current limitation of the VAC-Stent is its availability in a single size, while SEMS models are available in various lengths. This allows for the selection of the device on the basis of the anatomical location and size of the leak, which can be a crucial factor for tailored management [29].

Finally, the cost could be a significant limitation of the VAC-Stent. Currently, economic data are lacking, but the cost of the VAC-Stent is higher than that of SEMS and EVT. However, if future comparative studies demonstrate superior clinical efficacy, shorter hospitalization durations, and fewer required procedures, the overall treatment costs could potentially become comparable or even lower.

#### 3.4.2. VAC-Stent vs. EVT

##### Advantages

While EVT has firmly established itself as a viable alternative in ALs associated with fluid collections, the VAC-Stent offers a more complete solution by integrating the EVT sponge with an FC-SEMS [12,18,54]. This structure ensures drainage while preserving esophageal patency and applies radial force, potentially reducing the risk of luminal stenosis, a long-term complication associated with traditional EVT [18].

Unlike EVT, which often requires parenteral nutrition or the insertion of a nasogastric tube, patients with a VAC-Stent may resume oral feeding earlier, potentially improving their overall nutritional status and quality of life. However, further assessments are crucial regarding both management and dietary recommendations to optimize patient outcomes and prevent potential complications. Reports indicate that food particles can accumulate at the leak site after device removal or obstruct the external catheter of the device [60]. This underscores the importance of meticulous follow-up and dietary supervision to minimize the risk of post-procedural complications and ensure successful treatment outcomes.

EVT sponges generally require replacement every 3–5 days to mitigate risks such as tissue ingrowth and complications during removal, particularly for sponges placed intra-cavitary. This frequent replacement is crucial to prevent excessive integration of sponge material with surrounding tissues, which can complicate removal and increase the risk of further complications. Conversely, the VAC-Stent may remain in place for up to 7 days, reducing the frequency of procedures and associated discomfort for the patient. This extended interval between changes decreases physical and psychological stress on the patient and reduces the workload for healthcare providers. Moreover, it exposes the patient to a lower risk of AEs related to sedation and local complications (such as mucosal erosions, bleeding, and difficulties in sponge removal). Furthermore, the longer interval helps maintain a more stable internal environment, which can facilitate consistent healing, allowing for a gradual and controlled recovery process.

Finally, the VAC-Stent has the potential to be associated with fewer risks of AEs than EVT, especially in the intracavitary setting, which exerts negative pressures directly in the mediastinum with theoretically unpredictable consequences on nearby organs.

##### Disadvantages

The VAC-Stent has certain disadvantages compared with EVT. A significant advantage of EVT lies in its effective management of large dehiscences through intracavitary placement. This approach is critical, as it enables EVT to directly target the infectious focus, providing specific drainage and promoting the formation of granulation tissue, which facilitates gradual cavity healing [41]. The ability to customize the EVT sponge to fit the exact shape and size of the cavity is particularly beneficial, allowing for a tailored fit that enhances therapeutic efficacy [69].

Additionally, the more frequent scheduling of procedures with EVT allows for closer endoscopic reassessments, which are highly advantageous in monitoring the progress of treatment. These frequent assessments offer the flexibility to dynamically modify the treatment approach according to the evolving needs and responses of the patient.

One disadvantage of the VAC-Stent compared with EVT is that EVT can be custom-made at a significantly lower cost, reducing overall expenses for the device. This artisanal production of EVT devices offers a cost-effective alternative that the commercially produced VAC-Stent cannot match.

## 4. Conclusions and Future Perspectives

Despite significant advancements in surgery, ALs following esophagectomy remain a critical problem and a therapeutic challenge, with substantial morbidity and mortality. Over the years, a paradigm shift has occurred from surgical to endoscopic interventions.

The VAC-Stent represents the latest technological innovation in the endoscopic management of ALs. This device combines the advantages of SEMSs and EVT, offering a dual mechanism of action that excludes leaks and actively promotes healing through continuous aspiration of infected materials and enhancement of granulation tissue formation.

The VAC-Stent offers multiple benefits: it maintains the openness of the esophageal lumen, facilitates enteral nutrition, and serves as a drainage system for cavities linked to anastomotic dehiscence. Although the available data are still limited, they show promising results. Initial studies have demonstrated the VAC-Stent’s efficacy in treating post-esophagectomy Als and iatrogenic perforations, along with its utility as a rescue therapy in challenging cases where other methods have failed. Furthermore, the incidence of AEs is remarkably low.

In the broader context of endoscopic treatment for ALs, there is a notable deficiency in the literature concerning the comparative efficacy and safety of various treatment options. Currently, existing devices lack prospective studies with well-defined inclusion criteria and robust methodologies. This research gap also includes their cost-effectiveness—a critical factor considering the significant costs associated with prolonged medical treatments and potential hospital stays.

Future research should focus on studies that compare the VAC-Stent, EVT, and SEMSs, along with investigations into the optimal indications for each method. Identifying which types of leaks—regarding their etiology, anatomical location, size, and timing—are best managed with each device could lead to more customized and effective treatment strategies. Additionally, exploring the potential for combined treatments, whether sequential or concurrent, and defining the protocol for such strategies is imperative.

The development of a comprehensive treatment algorithm for ALs is critically needed. Such an algorithm would provide a systematic approach to managing these complex conditions, integrating the expertise of multidisciplinary teams including surgeons, radiologists, and gastroenterologists. By tailoring endoscopic treatments to align with the specific clinical and anatomical characteristics of each patient, outcomes would likely improve, thereby enhancing the quality of life for patients undergoing esophagectomy.

In this algorithm, there is potential for incorporating the VAC-Stent as a preferred option for specific conditions where its unique properties can be most beneficial. This would allow for the strategic use of the VAC-Stent, adapting its application on the basis of individual patient needs and specific clinical scenarios.

## Figures and Tables

**Table 1:** VAC-Stent: efficacy and safety data.

First Author (Publication Year)	Study Design	N° Patients N° of VAC-Stent (Mean per Patient)	Indications	Previous ET	Clinical Success	Rescue Treatments	Adverse Events
L. M. D. Pattynama et al. (2023) [21]	Prospective	10 patients 15 VAC-Stents	Post-esophagectomy AL (8 cases) Boerhaave syndrome (1 case) Iatrogenic perforation (1 case)	EVT (6 cases—60%)	10 patients (100%)	None	Anastomotic stricture (1 case undergone previous EVT)
J. Lange et al. (2023) [56]	Prospective	15 patients 41 VAC-Stents (2.7 per patient)	Post-esophagectomy ALs (11 cases) Iatrogenic perforation (3 cases) LINX band explantation (1 case)	EVT (7 cases—47%)	12 patients (80%)	Surgery (2 cases)	Dislocation (3 cases—7%) Mucosal erosion (9 cases—22%) Local bleeding (5 cases, 12%) Anastomotic stricture (1 case, 6.7%)
J. Lange et al. (2021) [57]	Retrospective	3 patients 4 VAC-Stents (1.3 per patient)	Post-esophagectomy AL (1 case) Boerhaave syndrome (1 case) Iatrogenic perforation (1 case)	SEMS (1 case) EVT (1 case)	3 patients (100%)	None	None
J. Lange et al. (2023) [58]	Prospective	9 patients 11 VAC-Stents (1.2 per patient)	Pre-emptive (9 cases)	None	8 cases did not develop AL	None	None
L. M. D. Pattynama et al. (2023) [59]	Case report	1 patient 1 VAC-Stent	Boerhaave syndrome (1 case)	Surgery, EVT	1 patient (100%)	None	None
S.H. Chon et al. (2022) [60]	Prospective	20 patients 24 VAC-Stents (1.2 per patient)	Post-esophagectomy AL (18 cases) Iatrogenic perforation (2 cases)	EVT (3 cases—15%)	12 out of 20 (60%) Primary treatment: 12 out of 17 (71%), rescue treatment: 0 out of 3 (0%)	EVT (7 cases) Surgery (1 case)	None
S.H. Chon, et al., (2021) [61]	Retrospective	10 patients 15 VAC-Stents (1.5 per patient)	Post-esophagectomy AL (5 cases) Iatrogenic perforation (1 case) Boerhaave syndrome (2 cases) Esophageal fistula (2 cases)	SEMS (1 case—10%), EVT (2 cases—20%) OTSC (2 cases—20%)	7 out of 10 (70%). Primary treatment: 4 out of 5 (80%), rescue treatment: 3 out of 5 (60%)	EVT (3 cases) Surgery (1 case)	Adherence to the oesophageal wall during stent removal (3 cases—30%)
J. Shah et al. (2023) [63]	Case report	1 patient 1 VAC-Stent	Esopleural fistula with empyema following a Roux-en-Y gastric bypass	SEMS and double pigtail stents	1 patient (100%)	None	None
K. Basiliya et al. (2024) [64]	Case report	1 patient/1 VAC-Stent	Anastomotic leak (1; colo-colonic anastomosis)	None	1 patient (100%)	None	None
S. H. Chon et al. (2020) [65]	Case report	1 patient 2 VAC-Stents	Post-gastrectomy anastomotic leak	OTSC	1 patient (100%)	None	None

SEMS: Self-expanding metal stents; EVT: endoscopic vacuum therapy; OTSC: over-the-scope clip, AL: anastomotic leak, ET: endoscopic treatment.

**Table 2:** VAC-Stent advantages and disadvantages compared with sponge EVT and SEMS.

	VAC-Stent versus SEMSs	VAC-Stent versus EVT
Advantages	Vacuum therapy (drainage and aspiration of fluid collection) Greater suitability for the esophageal lumen (less mucosal/vessel trauma) Lower risk of migration Lower stent leakage Rescue drainage strategy Sequential vacuum therapy after intracavitary EVT	Drainage capability associated with stent radial force (less risk of strictures) Resumption of oral feeding Slightly more spaced-out endoscopic procedures (every 5–7 days) Lower risk of AEs related to negative pressure directly in the mediastinum
Disadvantages	Dedicated training for endoscopists/nurses Need for hospitalization and monitoring Need for device replacement (every 5–7 days) Discomfort due to nasal tube Only one size Higher costs	Intracavitary placement for ALs associated with cavities not allowed Slightly delayed endoscopic re-evaluation for ALs No possibility for custom-made device Higher costs

SEMSs: Self-expanding metal stents, EVT: endoscopic vacuum therapy, ALs: anastomotic leaks.

## Data Availability

No new data were created.
